# Supplementary material for: Patterns of Intron Gain and Loss in Fungi
Source: PLoS Biol. 2004 Nov 30;2(12):e422. doi: 10.1371/journal.pbio.0020422 (PMC532390; doi:10.1371/journal.pbio.0020422)
Supplement: Table S1 — Also available at http://genes.mit.edu/NielsenEtAl/. (4.3 MB ZIP). [file pbio.0020422.st001.zip › NielsenEtAl/html/1192.html]

AN8176.1.NCU03757.1.MG08190.1.FG07186.1


```
 CLUSTAL W (1.82) Multiple Sequence Alignments - Introns Inserted


Sequence 1: NCU03757.1	361 aa
Sequence 2: MG08190.1	371 aa
Sequence 3: FG07186.1	352 aa
Sequence 4: AN8176.1	372 aa
Alignment Length: 372 aa
Number Identitical Residues: 218 aa
Alignment Score (without introns) 10026


MG08190.1 	MASRPTVTIIGADGKASGATHPIPNVFTAPIRPDIVQ~QVHKGMAKNKRQPYAVSEKAGH
NCU03757.1	MASRPTVTIIGADGKATGTTEVLPKVFGAPIRPDIVK~HVHTGMAKNKRQPYSVSEKAGH
FG07186.1 	MASRPTVTILGKDGAPTGATHAIPAVFTSPIRPDIVQ~QVHTGMAKNKRQPYSVSEKAGH
AN8176.1  	MASRPTVSIASGEGKPTGATCPLPAVFNSPIRPDIVQ2QVHTGMAKNKRQPYAVSEKAGE
          	*******:* . :* .:*:*  :* ** :*******: :**.**********:******.

MG08190.1 	QTSAESWGTG1RAVARIPR~VSGGGTHRAGQAAFGNMCRSGRMFAPTKVWRKWHIKVNQG
NCU03757.1	QTSAESWGTG1RAVARIPR~VSGGGTHRAGQAAFGNMCRSGRMFAPTKIWRKWHVKVNQG
FG07186.1 	QTSAESWGTG1RAVARIPR2VSGGGTHRAGQAAFGNMCRSGRMFAPTKIWRRWHIKVAVG
AN8176.1  	QTSAESWGTG1RAVARIPR~VSGGGTHRAGQAAFGNQCRSGRMFAPTKVWRKWHQKVNLG
          	********** ******** **************** ***********:**:** **  *

MG08190.1 	QK2RFATTSALAASAVAPLLLARGHRISNIPEVPLVIDSAAFSGAALAKTAAAVGLLNAV
NCU03757.1	QK2RFATASALAASAAAPLLMARGHQVSTVPEVPLVVDSAAFSG--VNKTANALALLKAV
FG07186.1 	QK2RYAVCSALAASAAVPLLQARGHQVNSVPEVPLVIDSAVFEAAAIAKTSAALGLLKAV
AN8176.1  	QR~RFATASALAASSVPSLLFARGHRIANVPEVPLVVDSKTFENAAVTKTKAAVALLQAL
          	*: *:*. ******:. .** ****:: .:******:** .*. ::: **  *:.**:*:

MG08190.1 	GAGADLDKVKKSRAMRAGKGKLRGRRYRQRRGPLVIYDPATDGTDLVKGFRNIPGVETSS
NCU03757.1	GAGADVQKAKASKKLRAGKGKLRDRRHRQRRGPLVVYSAETDGKDLIKGFRNIPGVETSP
FG07186.1 	GAGEDVSKVKNSKKIRAGKGKLRGRRHRQRRGPLVIYSPETDGKELVTAFRNITGVETCP
AN8176.1  	GAGPELVKVSKSRKLRAGKGKLRDRRFRQRRGPLVVYNPEVDGKELVRAFRNIPGIETSP
          	*** :: *.. *: :********.**.********:*.. .**.:*: .****.*:**..

MG08190.1 	VYSLNLLQLAPGGHLGRFIVWTSAGFKALDNIYGTTTTPSALKKDFLLPSNVVSQADLTR
NCU03757.1	VDALNLLQLAPGGHLGRFIVWTSAAIKELDAVYES-------KKGFQLPANVVAQADLTR
FG07186.1 	VTALNLLQLAPGGHLGRFIVWTSGAFKALDSIYGSTTEASAHKRDFLLPSNVVSQADLTR
AN8176.1  	VFSLNLLQLAPGGHLGRFIVWTSSAFEALDTVYGTTSSPSALKKDYLLPSNLVANADLAR
          	* :********************..:: ** :* ::: .:: *:.: **:*:*::***:*

MG08190.1 	LINSSEIQSVLRAPKGTARTKRQGCLKKNPLRNKQILLRLNPYASAYQKNKLGEEKLREG
NCU03757.1	LINSSEIQSVLRTPKGDAKTKRSNVQKKNPLRNKQVLLRLNPYAATFAKEKLGEIKE-EG
FG07186.1 	LINSSEIQSSINAPKGDAITRRSAVQKKNPLKNKQVMLRLNPYATVFAQEAQKKQN----
AN8176.1  	LINSSEIQSVLRAPKGEARTKRANVQKKNPLRNKQVMLRLNPYAAAFSKEKLGQKGVESE
          	********* :.:*** * *:*    *****:***::*******:.: ::   :    . 

MG08190.1 	KPE-VPEAFKTQLHEN
NCU03757.1	KPQRVPATFKTLLQE-
FG07186.1 	----------------
AN8176.1  	KPAPLADGFLKTLHEE
          	..   .    .   ..
```
